# Prediction of post-earthquake depressive and anxiety symptoms: a longitudinal resting-state fMRI study

**DOI:** 10.1038/srep06423

**Published:** 2014-09-19

**Authors:** Jinyi Long, Xiaoqi Huang, Yi Liao, Xinyu Hu, Junmei Hu, Su Lui, Rui Zhang, Yuanqing Li, Qiyong Gong

**Affiliations:** 1 Center for Brain Computer Interfaces and Brain Information Processing, South China University of Technology, Guangzhou 510640, China; 2 Huaxi MR Research Center, Department of Radiology, State Key Laboratory of Biotherapy, West China Hospital of Sichuan University, Chengdu 610041, China; 3 School of Basic Science and Forensic Medicine of Sichuan University, Chengdu, 610041, China; 4 These authors contributed equally to this work.

## Abstract

Neurobiological markers of stress symptom progression for healthy survivors from a disaster (e.g., an earthquake) would greatly help with early intervention to prevent the development of stress-related disorders. However, the relationship between the neurobiological alterations and the symptom progression over time is unclear. Here, we examined 44 healthy survivors of the Wenchuan earthquake in China in a longitudinal resting-state fMRI study to observe the alterations of brain functions related to depressive or anxiety symptom progression. Using multi-variate pattern analysis to the fMRI data, we successfully predicted the depressive or anxiety symptom severity for these survivors in short- (25 days) and long-term (2 years) and the symptom severity changes over time. Several brain areas (e.g., the frontolimbic and striatal areas) and the functional connectivities located within the fronto-striato-thalamic and default-mode networks were found to be correlated with the symptom progression and might play important roles in the adaptation to trauma.

A severe 8.0 magnitude earthquake occurred in Wenchuan, Sichuan, China, on May 12, 2008. Although only a minority of the survivors are likely to develop stress-related disorders such as acute stress disorder (ASD) and post-traumatic stress disorder (PTSD), psychological support may be required for those without stress-related disorders^1^, mainly because these healthy survivors are in a high risk for chronic PTSD or other mental problems such as depressive and anxiety disorders^2^.

Both alterations in brain structure and function after the disaster had been previously evaluated by magnetic resonance imaging (MRI)^3^,^4^,^5^. Furthermore, the longitudinal study of brain structure and function associated with the progression of symptom severity might play an important role in the exploration of related brain mechanisms and the development of effective biomarkers^6^. For example, one previous longitudinal study of structural MRI examined trauma-exposed survivors and found that greater cortical thickness in the dorsolateral prefrontal cortex was associated with greater PTSD symptom reduction^4^. Another longitudinal study using the structural MRI to examine the post-earthquake subjects without PTSD found that the PTSD symptoms were negatively associated to the regional grey matter volume (rGMV) in the right ventral anterior cingulate cortex (ACC) before the earthquake, and the decreased rGMV in the left orbitofrontal cortex (OFC) through the earthquake^5^. Two task-fMRI studies on PTSD revealed that symptom severity improvement was correlated with alterations of brain functions in certain areas, such as the ACC^7^,^8^. Using resting state fMRI (rs-fMRI), past studies have shown that the current or future severity of PTSD symptoms is associated with alterations of several brain areas, including the amygdala, medial prefrontal cortex (mPFC), and hippocampus^3^,^7^, or networks including the connectivity between the posterior cingulate cortex (PCC) and the bilateral amygdala^9^,^10^.

However, the relationship between longitudinal cerebral changes and symptom severity progression in trauma exposers remains to be established. Although the trauma exposers, especially those without PTSD, may lack structural brain change shortly after the trauma, brain functional changes had been demonstrated as short as within just one month of the earthquake^3^. Thus, we focused on the rs-fMRI in the current study to explore the relationship between longitudinal cerebral changes and symptom severity progression for trauma survivors not yet diagnosed with PTSD.

The amplitude of low-frequency fluctuation (ALFF) of the BOLD signal is one of the two ways most often used in rs-fMRI data analysis because it can provide information regarding synchronous regional cerebral activity^11^. In addition to regional ALFF, functional connectivity (FC) analysis allows the integrity of distributed brain networks to be examined and it is another commonly used method in rs-fMRI^3^,^12^. Since ALFF and FC reflected different domain of brain functional activities, both altered regional brain functions and altered FCs should be simultaneously monitored during the evolution of stress-related disorders.

In the present study, we first hypothesized that alterations of brain functional activities including ALFF and FC calculated from rs-fMRI could be used to classify people exposed to trauma who did not exhibit any overt stress-related disorders from controls and furthermore, they could also be used to predict current stress-related depressive and anxiety symptom severity. Our second hypothesis is that longitudinal alterations in rs-fMRI after trauma might be used to predict the progression of depressive and anxiety symptom. We tested our hypotheses by applying a multi-variate pattern analysis (MVPA) method, which is a data-driven technique that does not require any anatomical hypothesis regarding the functional localization of relevant brain processes, to longitudinal rs-fMRI data collected from survivors of the Wenchuan earthquake.

## Results

### Classification results

**Figure 1:**
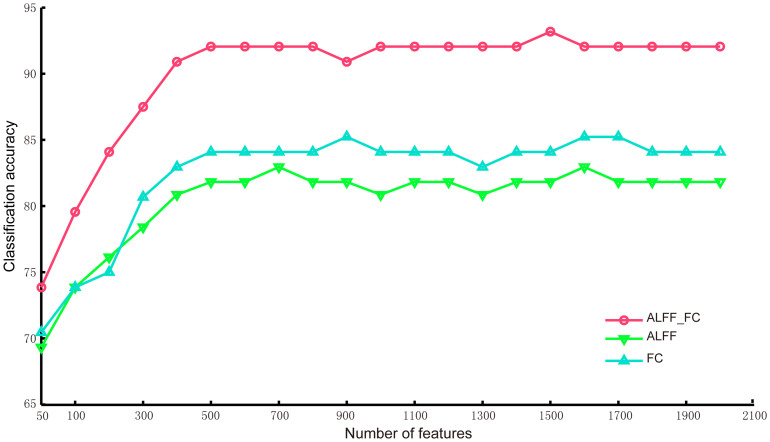
Classification accuracy curves with respect to the number of selected ALFF or FC features. Each curve corresponds to a feature type (hybrid features of ALFF and FC, ALFF-only or FC-only) for the classification of survivors between healthy controls at Time 1.

The classification accuracy curves for the data collected at time 1 with respect to the number of selected features (hybrid features of ALFF and FC, ALFF-only and FC-only features) are shown in Figure 1. These curves illustrate that the accuracies were close to be stable when the number of features was larger than 500. Therefore, we used 500 selected ALFF and FC features to present the detailed classification results.

The classification accuracy using the hybrid features of ALFF and FC for discriminating individuals as either controls or survivors at Time 1 was 92.05% (permutation test, *p*<0.001; 90.19% sensitive; 93.18% specific). The classification accuracy rate was 81.82% using only the ALFF features (permutation test, *p*<0.001; 75% sensitive; 81.82% specific), whereas the classification accuracy rate was 84.09% using only the FC features (permutation test, *p*<0.001; 81.82% sensitive; 86.36% specific). Thus, the classification performance using both the ALFF and FC features was higher than that using only the ALFF or FC features.

### Prediction results

#### Prediction performance

**Figure 2:**
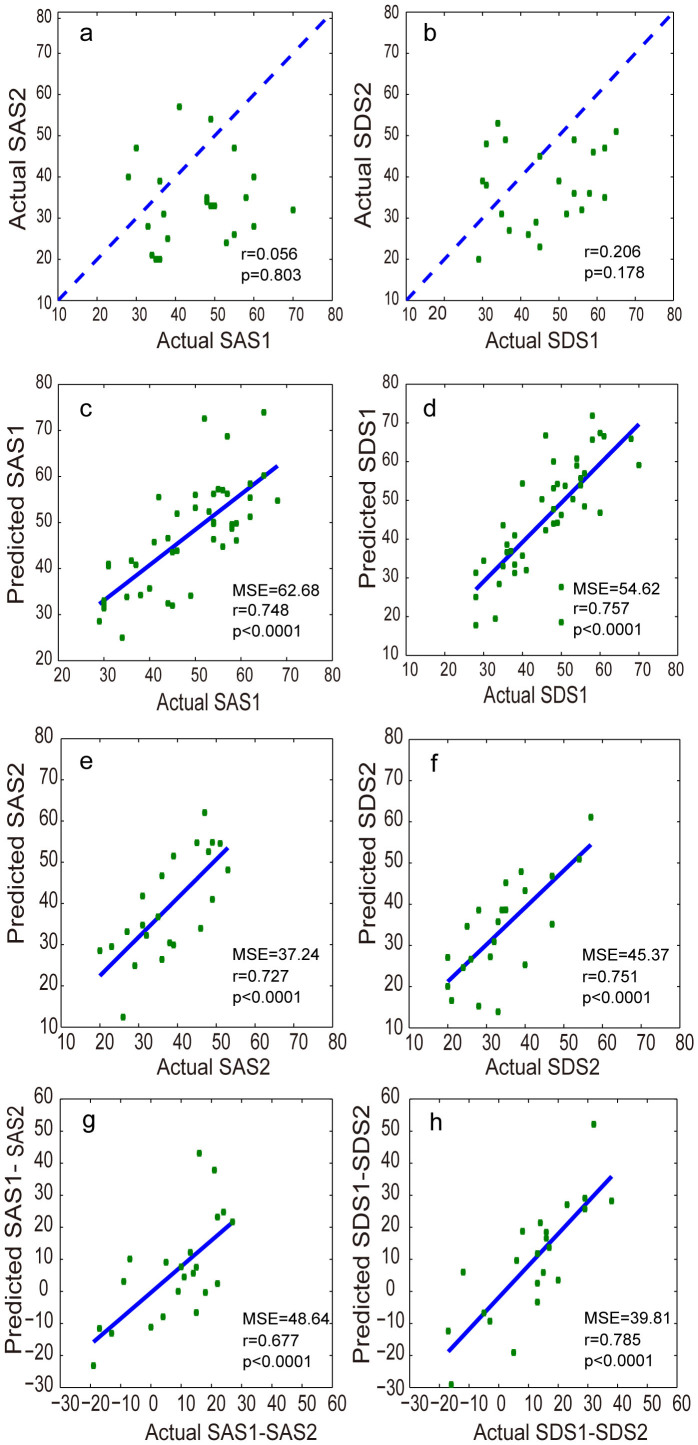
(a–b) Scatterplots depicting the relationship between the SAS (left) and SDS scores (right) at Times 1 and 2. The dashed lines represent 45°, which implies no change between Times 1 and 2. (c–f) Scatterplots depicting the correlation between the predicted SAS and actual SAS scores at Times 1 (c) and 2 (e) and between the predicted SDS and actual SDS scores at Times 1 (d) and 2 (f). (g–h) Scatterplots depicting the correlation between the difference in the predicted SAS scores at Times 1 and 2 (the predicted SAS1-SAS2) and the difference in the actual SAS scores at Times 1 and 2 (the actual SAS1-SAS2) (g) and the correlation between the difference in the predicted SDS scores at Times 1 and 2 (the predicted SDS1-SDS2) and the difference in the actual SDS scores at Times 1 and 2 (the actual SDS1-SDS2) (h).

The support vector regression (SVR) model predicted the true Self-Rating Anxiety Scale (SAS)/Self-Rating Depression Scale (SDS) scores with high/significant accuracy at Time 1 (permutation test, both *p*<0.001) and Time 2 (permutation test, both *p*<0.001) respectively. Figure 2 a–b shows that there was no significant correlation between the actual SAS/SDS scores at Time 1 and those at Time 2, but the SAS/SDS scores at Time 1 were significant higher than those at Time 2 (both *p*<0.05). Figure 2 c–f and Table 1 shows that there was a significant positive correlation between the predicted and actual SAS/SDS scores at both Time 1 and Time 2.

We also computed the average correlation coefficients between the SAS/SDS obtained in the permutation test and the actual SAS/SDS at Time 1 (r = −0.026 ± 0.031/r = 0.037 ± 0.023) and Time 2 (r = 0.048 ± 0.052/r = 0.022 ± 0.041), which were close to zero (both *p*>0.05) and reflected the effectiveness of our predictions.

In addition, Figure 2 g–h shows that there was a significant positive correlation between the change of predicted symptom severity at Times 1 and 2 (i.e., the predicted SAS1-SAS2 or predicted SDS1-SDS2) and the change in the actual symptom severity at Times 1 and 2 (i.e., the actual SAS1-SAS2 or actual SDS1-SDS2) (see Table 1). Table 1 also shows that the predicted values of SAS1-SAS2 or SDS1-SDS2 were more highly correlated within measures than across measures.

#### The spatial distribution of informative voxels for SAS/SDS prediction

**Figure 3:**
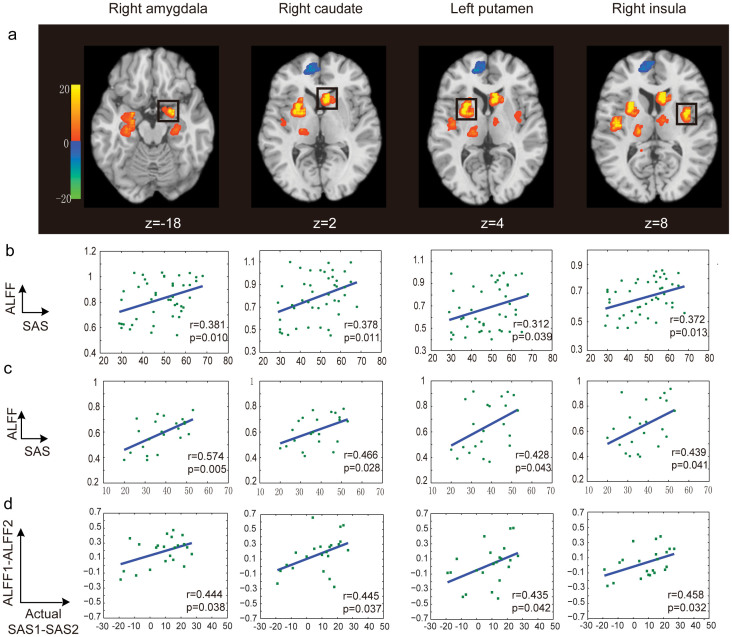
The distribution of the most informative voxels in the SAS prediction. (a) The SVR weight map: the color intensities indicate the signed SVR weights. Brain areas showed significant correlations between the ALFF features and SAS are indicated by rectangles. There are significant positive correlations between the ALFF features and SAS at Time 1 (b) and Time 2 (c) for each indicated brain area. And there are also significant positive correlations of the changes of ALFF and the alterations of the actual symptom severity of SAS between Time 1 and Time 2 (d). Note: **p<0.01, t-test. No brain region was found, of which the ALFF features were significantly negatively correlated with SAS at Times 1 and 2.

**Figure 4:**
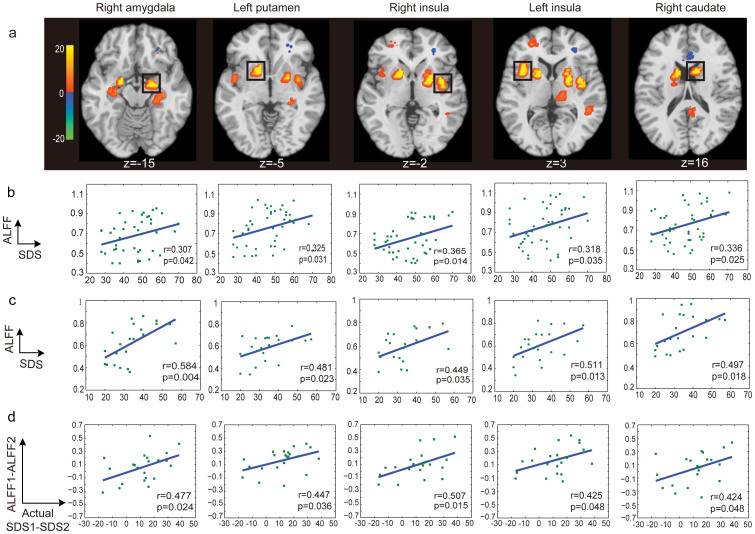
The distribution of the most informative voxels in SDS prediction. (a) The SVR weight map: The color intensities indicate the signed SVR weights. Brain areas showed significant correlations between the ALFF features and SDS are indicated by rectangles. There are significant positive correlations between the ALFF features and SDS at Time 1 (b) and Time 2 (c) for each indicated brain area. And there are also significant positive correlations of the changes of ALFF and the alterations of the actual symptom severity of SDS between Time 1 and Time 2 (d). Note: **p<0.01, t-test. No brain region was found, of which the ALFF features were significantly negatively correlated with SDS at Times 1 and 2.

The spatial distributions of the informative voxels for SAS and SDS prediction are shown in Figures 3a and 4a, with different colors representing the SVR weights of ALFF features. We found that the voxels with greater predictive power for both SAS and SDS were located in brain regions including the precentral gyrus, prefrontal cortex, caudate, insula, putamen, hippocampus, amygdala and ACC.

As shown in Figure 3b/4b, we identified several brain regions that showed a significant positive correlation with SAS/SDS at Time 1, such as the right amygdala, right caudate, right insula and left putamen. There was also a significant positive correlation between the ALFF features of these brain regions and SAS/SDS at Time 2 (Figure 3c/4c). Furthermore, Figure 3D/4D shows that the changes of ALFF in these brain regions were also significantly positively correlated with the changes in the actual symptom severity of SAS/SDS between Time 1 and Time 2. We did not find any brain region of which the ALFF features were significantly negatively correlated with SAS/SDS at Times 1 and 2.

#### Functional connectivity with high discriminative power for SAS/SDS prediction

**Figure 5:**
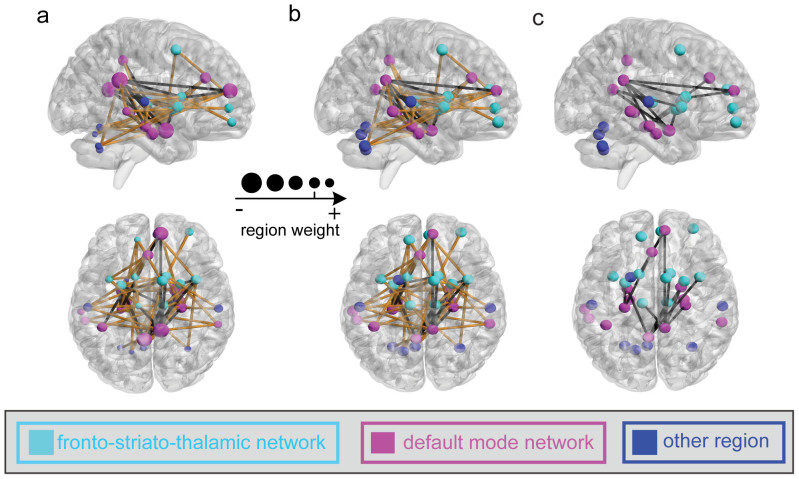
The distribution of the most informative FCs. (a) and (b): for SAS prediction at Time 1 and Time 2, respectively. The line color represents the significance of the correlation between the FC values and the SAS (black: significant negative correlation with p<0.05; yellow: insignificant correlation with p>0.05; no significant positive correlation was found). (c): the FCs with significant negative correlation of the changes of FC and the alterations of the actual symptom severity of SAS between Time 1 and Time 2. Regions indicated by the nodes are colors-coded by category (cyan: fronto-striato-thalamic network; magenta: default-mode network; blue: other region); and for (a), the radius of each node is scaled with the sum of the corresponding SVR weights of all the connections to and from that region.

**Figure 6:**
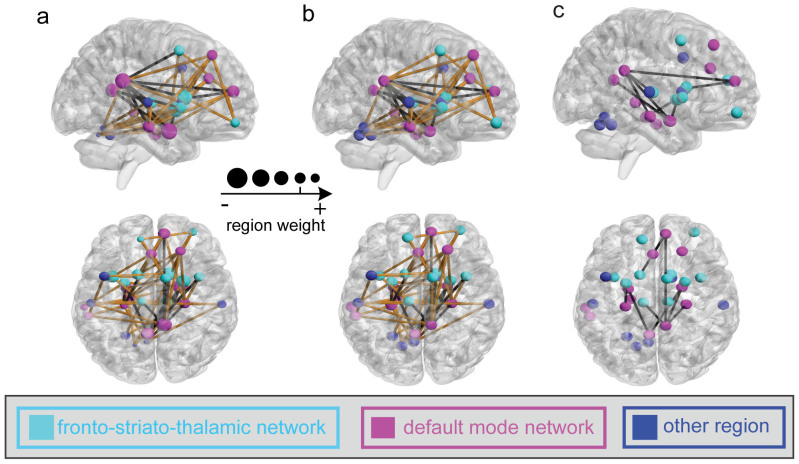
The distribution of the most informative FCs. (a) and (b): for SDS prediction at Time 1 and Time 2, respectively. The line color represents the significance of the correlation between the FC values and the SDS (black: significant negative correlation with p<0.05; yellow: insignificant correlation with *p*>0.05; no significant positive correlation was found). (c): the FCs with significant negative correlation of the changes of FC and the alterations of the actual symptom severity of SDS between Time 1 and Time 2. Regions indicated by the nodes are colors-coded by category (cyan: fronto-striato-thalamic network; magenta: default-mode network; blue: other region); and for (a), the radius of each node is scaled with the sum of the corresponding SVR weights of all the connections to and from that region.

In this investigation, we observed 85 and 80 most informative FCs for SAS and SDS predictions, respectively, at Time 1 through a permutation test at *p*<0.05 with a family-wise error (FWE) correction procedure, as shown in Figures 5a and 6a. Two networks including the fronto-striato-thalamic network and the default-mode network were identified for both SAS and SDS predictions. We found that most of the brain regions had negative weights for the prediction and several brain regions exhibited more negative weights than others: i.e., the bilateral amygdala, right putamen, right hippocampus, bilateral insula, right caudate, and left ACC, which were mainly located within the default-mode network.

Furthermore, based on the data in Time 1, these most informative FCs exhibited two classes: (i) their strengths were significantly negatively correlated with SAS or SDS (*p*<0.05, black lines in Figures 5a and 6a) and (ii) their strengths were not significantly correlated with SAS or SDS (*p*>0.05, yellow lines in Figures 5a and 6a). No informative FCs were found to show significant positive correlation with SAS or SDS. The FCs weakening with respect to SAS or SDS were mainly located within the default-mode network. Similar correlation results were obtained using the data at Time 2 (Figures 5b and 6b). Furthermore, Figure 5c/6c showed the distribution of the FCs with significant negative correlation between the changes of FC and the alterations of the actual symptom severity of SAS/SDS between Time 1 and Time 2.

## Discussion

In the present longitudinal rs-fMRI study, we investigated the potential alterations of brain functions related to the severity of current depressive or anxiety symptoms and their changes over time, which began after the 2008 Wenchuan earthquake in China. By applying a sparse MVPA method to the rs-fMRI data, we successfully discriminated the survivors of an earthquake from controls with 92.05% accuracy at Time 1, and predicted depressive or anxiety symptom severity at both Times 1 and 2 and its changes over time (*p*<0.001). Moreover, we demonstrated that the performance of the classification and prediction could be improved using the hybrid features of ALFF and FC. The most informative voxels for symptom severity prediction were mainly located in the mPFC, pre-frontal limbic system, pre-SMA, and striatal system, which are associated with emotion processing and memory^13^,^14^. Furthermore, the informative FCs were mainly located within the fronto-striato-thalamic network and default-mode network.

In this study, we first demonstrated the effectiveness of the MVPA method for the detection of mental disorder-risk group. Neural signatures of stress and anxiety are often quite subtle, and reside only in a very small set of discreet circuits^3^,^15^. This challenges the effective detection of mental disorder-risk group, as well as the prediction of changes in their symptom severity over time. This problem may be surmounted to some degree by a methodological shift to an MVPA approach, which can boost sensitivity by pooling the contributions of multiple voxels, including those with and without significant responses to any of the conditions of interest^16^. Several brain imaging studies have applied MVPA methods to distinguish psychiatric patients (e.g., depressive disorders and Alzheimer's disease) from healthy controls^11^,^12^. In particular, using an MVPA method, Koutsouleris et al. obtained an accuracy of over 80% for differentiating patients with prodromal symptoms of schizophrenia from controls^17^. Our results also suggested that the MVPA approach could be used to perform classification between mental disorder-risk and control groups and to make predictions about depressive or anxiety symptom severity for healthy survivors of earthquakes. Furthermore, compared to the voxels obtained with a univariate region of interest (ROI) method, those selected with an MVPA method could be more widely distributed as shown in Figures 3a and 4a.

The brain regions with significant predictive power for the prediction of current depressive or anxiety symptom severity were obtained by applying the MVPA method with a permutation test to the data collected at Time 1, where the predictive power was assessed using the SVR weights of the ALFF features (see Materials and methods, Figures 3a and 4a). These most informative brain regions included the right amygdala, right caudate, right insula and left putamen, which showed a significant positive correlation with SAS/SDS at Time 1 (Figure 3b/4b) and Time 2 (Figure 3c/4c). These results are partially consistent with the findings from previous studies investigating the underlying brain regions related to the symptom severity of stress-related disorders. For instance, Lui et al.^3^ analyzed rs-fMRI data from earthquake survivors and healthy controls and observed a significant correlation between stress symptom severity and ALFF in several brain regions, including the putamen, amygdala, caudate, and hippocampus. Using a variety of tasks and stimuli for the recollection of traumatic memories and processing of fear and pain, several studies observed a significant correlation between the current depressive or anxiety symptom severity and the amygdala^18^,^19^ and mPFC activity^15^,^20^,^21^,^22^. Hopper et al.^23^ observed a positive correlation between the activation of the insula and symptom severity. The demonstration of involvement of similar regions in the current study, not only replicated the already known mechanism, but also shed light on the potential translational application of those intrinsic activity alterations.

Two main resting-state connectivity networks, the fronto-striato-thalamic and default-mode networks, identified with samples from Time 1, were shown to have significant predictive power for symptom severity at both Times 1 and 2 (Figures 2, 5, and 6). The fronto-striato-thalamic network contains the mPFC, caudate, putamen, insula, and thalamic cortex^24^, whereas the default-mode network includes the PCC, ACC, hippocampus, parahippocampus, and amygdala^25^. Both of these brain networks play important roles in the regulation of emotional processing^3^,^26^. By analyzing the SVR weights, and the correlations between the FC features and SAS/SDS at Times 1 and 2, we found that there were significant alterations of some FCs in the survivors. Specifically, the strengths of these FCs, which were mainly located in the default-mode network, showed a significant negative correlation with the post-traumatic depressive or anxiety symptoms. The other FCs mainly in the fronto-striato-thalamic network, which did not show significant alterations in the survivors, were also identified by the MVPA method and contributed to the SAS and SDS predictions. Previous studies suggested that these two brain networks are associated with stress-related symptom severity over time. Lanius et al.^9^ studied a group of acutely traumatized survivors six weeks after trauma and observed a correlation between post-traumatic stress symptoms and the strength of resting state connectivity of the PCC with the perigenual anterior cingulate and amygdala. Based on the rs-fMRI of the acutely traumatized survivors within six months post-accident, Zhou et al.^10^ observed that the strength of the resting-state connectivity between the PCC and the hippocampus/amygdala was related to the severity of PTSD symptoms. In addition, we revealed that not only were the informative FC features/strengths negatively correlated with the depressive or anxiety symptoms at Time 1 (Figures 5a and 6a), the changed FC features/strengths were also negatively correlated with the depressive or anxiety symptom severity at Time 2 (Figures 5b and 6b).

Furthermore, we found that the observed informative brain regions and FCs were involved in the evolution of stress symptom severity. In particular, the changes of the ALFF and FC were related to the changes of the actual symptom severity over time (Figures 3d, 4d, 5c, and 6c). This gave a strong support for the validation of our prediction model. Previous studies partially support our result. For instance, an association between post-traumatic stress symptom reduction through cognitive-behavioral therapy and the increased ACC activation and decreased amygdala activation was revealed by Felmingham et al.^8^. Furthermore, a longitudinal fMRI study of the neural correlates of the recovery from PTSD found a significant correlation between the degree of symptom improvement and activation changes within the hippocampus and subgenual ACC^7^. In addition, Lanius et al.^9^ observed a correlation between the connectivity strength of the posterior cingulate cortex/precuneus and right amygdala at six weeks post-trauma and the severity of the post-traumatic stress symptoms at both six and 12 weeks in a group of trauma survivors. Taken together, these findings suggested that the informative brain regions and networks involving the functional alterations with symptom changes might play important roles in the neurophysiology of stress adaptation.

Although several brain-imaging studies have explored the relationships between the alterations in brain function and post-traumatic stress symptom severity^3^,^15^,^18^; as far as we know, no studies have ever predicted depressive or anxiety symptom severity using imaging data. Using the SVR model trained at Time 1, we successfully predicted the symptom severity of survivors at Time 2 (*p*<0.001) in this study. We thus postulated that the relationship between the pattern of ALFF and FC extracted with the sparse MVPA method and the symptom severity might be stable over a long time. Our results also supported the observation that the dysfunctions of the underlying brain regions and FCs, which arise shortly after the traumatic event and can persist for years or even decades, correlate with symptom severity^3^,^15^,^18^,^19^,^22^. Interestingly, not only did we predict the symptom severity of samples at Time 2 with high performance based on those informative brain regions and FCs determined at Time 1 (Figure 2), we also observed the significant correlation with the depressive or anxiety symptom severity at Time 2 in those brain regions and FCs (Figures 3,4,5,6). Thus, we speculate that these brain regions and FCs might play a role in the neural rehabilitation process for psychological responses after a severe stress event, which might give a hint to the selection of future therapeutic target regions.

The main drawback of the present study is the relatively small sample size, particularly for Time 2. In addition, we were not able to observe a group of subjects who were scanned at Time 1 and subsequently developed PTSD. Hence, an expanded study with PTSD patients would be necessary to confirm whether this method can be used to predict the symptoms of psychiatric disorders. Finally, the present study focuses only on the rs-fMRI, while the MVPA method can also be applied to the structural MRI^27^. In the future, it might be worthwhile to apply this method to the structural MRI and finally to combine the structural and functional MRI together to explore whole profile of cerebral changes after big traumatic events so as to help with the intervention of those survivors.

## Methods

### Subjects

The participants in this study included physically healthy survivors of the Wenchuan earthquake (Mercalli intensity scale: 8.0) from the most affected regions and healthy controls from Chengdu city, which is 50 miles from the epicenter, who were unaffected by the earthquake. All participants provided written informed consent for their participation in the study. The survivors underwent resting-state fMRI scanning twice: 25 days (Time 1) after the earthquake and two years later (Time 2). Forty-four healthy survivors were recruited at Time 1, but only 22 of these participants were able to repeat the scanning at Time 2 because we lost contact with the others. The data collected at Time 1 are the same as those by Lui et al.^3^.

The inclusion criteria for all participants were described in our previously published paper^3^. In brief, the survivors at both time points underwent a structural Clinical Interview for the Diagnostic and Statistical Manual of Mental Disorders-IV (SCID) interview to rule out the past or current diagnosis of an Axis I disorder. Using a questionnaire developed in house, we excluded severe life events before the earthquake and a family history of psychiatric disorders for the survivors. For the survivors who attended the second scanning, no new severe life events were reported since the earthquake. The SAS^28^ and the SDS^29^ were used to evaluate the levels of anxiety and depression in the survivors at both time points, as shown in Table 2. The SAS and SDS are valuable in documenting and quantifying initial symptoms and complaints, as verified in a previous study^28^, and are easy to administer.

Forty-four healthy controls were recruited shortly before the earthquake for another study and were scanned only once using identical parameters^3^,^30^. All controls were screened using the SCID-non-patient version to confirm a lifetime absence of psychiatric illness and using a questionnaire developed in house to exclude severe life events and a family history of psychiatric disorders.

The demographic information of the participants is presented in Table 2 and illustrates that the 44 survivors and 44 healthy controls were matched with regard to age, gender, and years of education. There were no significant differences between the 22 follow-up survivors and the 22 survivors lost to follow-up in terms of age, gender, or years of education. All participants gave their written informed consent prior to the study, which was in accordance with the Declaration of Helsinki and approved by the local ethics committee.

### Resting experiment and data acquisition

A 3-T MR imaging system (EXCITE; General Electric) was used for scanning with a gradient-echo echo-planar imaging sequence. All MR imaging data acquisitions were conducted with the same equipment and procedure. The imaging parameters included the repetition time/echo time (2,000/30 ms), flip angle (90°), slice thickness (5 mm), matrix (64 × 64), field of view (FOV) (240 × 240 mm^2^), and voxel size (3.75 × 3.75 × 5 mm^3^). There were 30 axial slices in each brain volume. Each functional run contained 205 image volumes, corresponding to 410 s. In each rs-fMRI scanning session, the participants were required to close their eyes, relax, remain awake, and perform no specific cognitive tasks.

### Data preprocessing

All participants were allowed adequate head motion during the scan acquisition with <0.5 mm head translation movement and <0.5° rotation. The toolkit used for rs-fMRI data preprocessing and analysis included a statistical parametric mapping software package (SPM8, http://www.fil.ion.ucl.ac.uk/spm/) and the rs-fMRI Data Analysis Toolkit (REST, http://rest.restfmri.net)^31^. For each participant, only the final 200 volumes of scanned data were used for further data analysis (the initial five volumes, collected before magnetization equilibrium was reached, were discarded). The preprocessing steps included slice timing, realignment to the initial image, head-motion correction, normalization, and smoothing with a Gaussian filter of 4 mm full-width half-maximum kernel. During the normalization, each voxel was resampled to 3 × 3 × 3 mm^3^. The resulting images were detrended to abandon the linear trend and then temporally filtered with a Chebyshev band-pass filter (0.01–0.08 Hz).

### ALFF calculation

For the ALFF calculation, we used the REST software with a procedure similar to that in our previous study^3^. Briefly, a Fast Fourier Transform (FFT) was initially used to convert the time series from each voxel into the frequency domain. The square root power spectrum was then computed and averaged across 0.01–0.08 Hz at each voxel. The averaged square root power in this frequency band was used as the ALFF for this voxel. For each participant, the ALFF of each voxel was divided by the global mean ALFF value to reduce the global effect of variability across participants.

### Functional connectivity calculation

For the FC calculation, we initially created regions of interest (ROIs) for each participant by applying the free software WFU_PickAtlas (version 2.0, http://www.ansir.wfubmc.edu)^32^. According to the automated anatomical labeling atlas, we divided the fMRI volumes, registered with the Montreal Neurological Institute (MNI) template, into 116 regions^33^,^34^. Of the 116 regions, there were 90 regions in the cerebrum (45 in each hemisphere) and 26 in the cerebellum (nine in each cerebellar hemisphere and eight in the vermis). The fMRI signals over all voxels in each of the 116 regions were averaged to obtain the regional mean time series. Furthermore, for each regional mean time series, we regressed out the global mean, head motion, and confounding effects of cerebrospinal fluid (CSF) and white matter as well as the first-order derivative terms for the global, white matter, and CSF average signals^12^,^35^,^36^,^37^,^38^. Using the residuals of these regressions as the set of regional mean time series, we calculated the Pearson correlation coefficient between each pair of regions. This procedure resulted in a resting-state functional network described by a 116 × 116 symmetrical matrix for each participant. The FC features were spanned by the upper triangle elements of the FC matrix with 6670 dimensions.

### MVPA procedure

Based on the MVPA method, we first performed the classification with the samples of controls and the survivors of the earthquake at Time 1. Furthermore, we performed the prediction of the psychological symptom severity only with the samples of the survivors of the earthquake at both Times 1 and 2. Specifically, the MVPA for classification or prediction was performed by a leave-one-out cross-validation (LOOCV), as described in the following.

### 1) LOOCV for classification

In each fold of the LOOCV for classification, a sample from a control or a survivor at Time 1 was used for label prediction (test), and the remaining 87 samples including controls and survivors at Time 1 were used for selecting features and training the support vector machine (SVM) classifier (Spider Machine Learning Toolbox, http://www.kyb.tuebingen.mpg.de/de/bs/people/spider). After the LOOCV, the classification accuracy was determined as the ratio of the number of correctly classified samples to the total number of samples. The sensitivity represented the proportion of survivors been correctly classified, while the specificity represented the proportion of controls been correctly classified. We now explained the procedure of feature selection, model training, and test in one fold.

**Feature selection**. Feature selection was performed based on the training data set in this fold. Specifically, a sparse representation-based MVPA algorithm, developed in our previous paper^39^, was utilized for selecting informative features. Two types of features, i.e., ALFF and FC features, were used in the present study. We performed ALFF and FC feature selections separately. Firstly, we describe the sparse representation-based method for ALFF feature selection below.

*Initial selection of features based on a univariate method.* Because the number of ALFF features (i.e., the number of voxels) was too large, we initially reduced it to 4000 by applying a univariate method to the training data. The initial feature dimension reduction method was a two-sided t-test, through which we compared two different classes of training data (survivors vs. controls). We then selected the 4000 features with the highest absolute t-scores. Based on the initially selected 4000 ALFF features, the following sparse representation method was used for further feature selection.

*The sparse representation method for feature selection*^39^: A sparse representation-based feature selection algorithm was used to build a set of sparse coefficients corresponding to the features. The absolute values of these coefficiens represented the importance of the corresponding features, feature selection was thus performed based on these coefficients. Assume that the data matrix 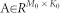 is a rs-fMRI feature matrix and that *M*_0_ and *K*_0_ are the number of subjects (87 subjects) and features (4000 ALFF feature values), respectively. Let 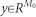 denote the vector of the sample labels (+1 for survivors in the earthquake and -1 for controls).

The algorithm contained *l*_0_ repeats of sparse representation (a bootstrapping processing). For the *k*th repeat, the data matrix *A_k_* was constructed by randomly extracting *L* rows from *A*. The data vector 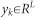 was formed with *L* entries of *y*. In this paper, we set *L* = 0.3*M*_0_ and *l*_0_ = 200. We then solved the following optimization problem, which could be converted to a standard linear programming problem^40^: 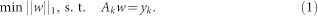
The optimal solution of (1) was denoted by 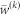, which was a weight vector of features.

After *l*_0_ repeats, we set 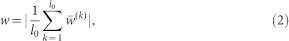
where *w* was a weight vector of features.

Using the weight vector *w*, we selected 500 features with the highest weights as in^39^.

Secondly, we also applied the above-described sparse representation-based method for the FC feature selection. Because the original number (i.e., 6670) of FC features was not very large, no initial feature selection/dimension reduction was used before the sparse representation-based feature selection. After the feature selection procedure based on the training data, 500 FC features were also selected for classification.

**Feature extraction***.* We concatenated the selected ALFF and FC features to construct a 1000-dimensional feature vector for each subject of the training data set and test data set.

**Model training**. Using the feature vectors of training data with labels (87 samples including controls and survivors at Time 1), a linear SVM was trained.

**Classification.** We applied the trained SVM to the test feature vector to determine whether this subject was a control or a survivor of the earthquake.

In the above LOOCV procedure, the dimension of feature vector was set to be 1000 (500 for ALFF features and 500 for FC features). We also systematically varied the number of selected ALFF and FC features from 50 to 2000 and performed classification for each dimension number.

### 2) LOOCV for prediction.

In each fold of the LOOCV for prediction, the fMRI data from one survivor at Time 1 were used for predicting the symptom level SAS/SDS (test), and the fMRI data and SAS/SDS scores from the remaining 43 survivors at Time 1 were used for selecting features and training a linear SVR model (Spider Machine Learning Toolbox, http://www.kyb.tuebingen.mpg.de/de/bs/people/spider). Then using the SVR model established at Time 1, we also predicted the symptom level at Time 2 whenever the data for the test survivor at Time 2 were available. After the LOOCV, the mean squared error (MSE) and the correlation coefficient between the predicted SAS/SDS and the actual SAS/SDS for all samples were used as the prediction performance indices. We explained the detailed procedure of feature selection, model training, and test in one fold as below.

**Feature selection**. The feature selection based on the training data set was similar to that for the above classification except for the initial selection of features. First, for the initial selection of ALFF features, we computed the correlation coefficient between the feature values of the training samples in each dimension and the corresponding actual symptom severity scores (SAS/SDS) and selected the 4000 features with the largest absolute values of the correlation coefficients. Same as for the classification, we further selected 500 from the initially selected 4000 ALFF features using the above sparse representation method. Note that the rs-fMRI feature matrix A here was 43 by 4000 dimensional, and 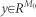 was the vector of symptom severity scores (SAS/SDS). Next, we applied the sparse representation-based method to the training data and selected 500 FC features.

**Feature extraction.** We concatenated the selected ALFF and FC features to construct a 1000-dimensional feature vector for each subject of the training data set and test data set. If the data at Time 2 from the test survivor were available, we also extracted the corresponding 1000-dimensional feature vector based on the selected ALFF and FC features.

**Model training.** Using the feature vectors of the training data (43 samples at Time 1) with their corresponding levels of symptom severity (i.e., SAS or SDS), a linear SVR was trained.

**Prediction.** We applied the trained SVR model to the test feature vector at Time 1 to predict the level of symptom severity (SAS/SDS) of the corresponding survivor (test sample at Time 1).

In this fold of LOOCV, if the data at Time 2 from the test survivor were available, then the established SVR model was also used to predict the level of symptom severity (SAS/SDS) for this sample at Time 2. Furthermore, we calculated the difference of the test feature vectors at Time 1 and Time 2. By applying the SVR model to the difference vector, we predicted the symptom severity change over time for this test survivor. Because the SVR model is linear, the predicted symptom severity change over time was equivalent to the difference between the predicted SAS/SDS at Time 1 and the predicted SAS/SDS at Time 2.

### Permutation tests

In this study, we used non-parametric permutation tests to assess the statistical significance of all the LOOCV results (i.e., SVM classification accuracy rates and SVR prediction results) and to determine the most informative features^12^,^41^.

### 1) Significance test of classification accuracy

We performed 1000 permutations as a significance test of classification accuracy. In each permutation, we randomly assigned the subjects' class labels and performed the above-described MVPA procedure, including the feature selection and classification, to compute classification accuracy. Finally, a null distribution composed of 1000 accuracy rates corresponding to the 1000 permutations was obtained. The *p*-value was estimated as the proportion of the accuracies in the null distribution that were greater than or equal to the actual classification accuracy, which was obtained using the non-permutated training data.

### 2) Significance test of prediction result

We also performed 1000 permutations for the SVR prediction of symptom severity. In each permutation, the stress scores (i.e., SAS or SDS) were randomly assigned to the subjects, and the above-described MVPA procedure, including feature selection and prediction, was performed to obtain a correlation coefficient. Finally, a null distribution composed of the 1000 correlation coefficients corresponding to the 1000 permutations was constructed. The *p*-value was estimated as the proportion of the correlation coefficient values in the null distribution that were greater than or equal to the actual correlation coefficient obtained using the non-permutated training data.

### 3) Informative features identification

Note that the absolute weight of each feature determines its importance in the prediction regarding the levels of emotional distress^42^. Hence, our procedure for the identification of informative ALFF and FC features was based on the absolute weights of features obtained through SVR model training. In each fold of the LOOCV for the SAS prediction, 500 ALFF and 500 FC features were selected through the sparse representation-based MVPA algorithm, and their weights were obtained. The absolute values of the 500 ALFF weights and 500 FC weights were normalized to [0, 1] and then used to construct two weight maps for the ALFF features and the FC features, respectively (the ALFF features that were not initially selected were assigned a weight of 0). Finally, an average weight map for the ALFF or FC features was obtained by averaging the above weight maps across all folds.

Next, we determined a threshold to identify the most informative ALFF or FC features through a permutation test. After 1000 permutations for the symptom severity SAS prediction as described above, we obtained 1000 average weight maps for the ALFF features and 1000 average weight maps for the FC features. For multiple comparison correction, an FWE correction procedure was conducted as described below. The maximum voxel weight was identified for each average weight map, and a null distribution was constructed using these maximum voxel weights for the ALFF or FC features^43^. Then, a weight threshold corresponding to *p* = 0.05 (FWE corrected) was obtained from the null distribution of ALFF or FC features. Using this threshold, we identified the significantly informative ALFF or FC features from the corresponding actual average weight map. For the informative FCs, we assessed the network affiliation of their nodes or brain regions according to previous results^24^,^25^.

For the SDS prediction, we performed a similar procedure as above to identify the informative ALFF and FC features.

## Author Contributions

Y.L., X.Y.H. and J.M.H. conducted the clinical testing of participants, X.Q.H., Y.L. and S.L. collected the data, J.Y.L., R.Z. and Y.Q.L. analyzed the data, and, J.Y.L., X.Q.H., Y.Q.L. and Q.Y.G. wrote the manuscript.

## Figures and Tables

**Table 1:** The correlation coefficients between the predicted and actual symptom levels

	Actual SAS (Time 1/2)	Actual SDS (Time 1/2)	Actual SAS1-SAS2	Actual SDS1-SDS2
Predicted SAS (Time 1/2)	0.748/0.727	0.574/0.548		
Predicted SDS (Time 1/2)	0.621/0.569	0.757/0.751		
Predicted SAS1-SAS2			0.677	0.508
Predicted SDS1-SDS2			0.462	0.785

**Table 2:** Demographic information and clinical characteristics of the participants in this study

	Survivors (Mean ± SD)		Controls (Mean ± SD)	*p-*value
Time 1				
Sample size	44		44	—
Gender (male/female)	27/17		25/19	0.664^a^
Age (years)	37 ± 10.6		35.8 ± 12.2	0.521^b^
Education (years)	8.6 ± 4.1		9.8 ± 4.6	0.432^b^
Days after earthquake	21 ± 3		—	—
SAS	48.4 ± 11.4		—	—
SDS	46.8 ± 10.8		—	—
Time 2				
	Follow up subjects	Subjects lost		
Sample size	22	22	—	—
Gender (male/female)	13/9	14/8	—	0.724^a^
Age (years)	39.3 ± 10.2	38.7 ± 11.5	—	0.476^b^
Education (years)	8.8 ± 5.6	8.4 ± 4.7	—	0.536^b^
Days after earthquake	753 ± 28	—	—	—
SAS	34.1 ± 10.2	—	—	—
SDS	37.7 ± 9.7	—	—	—

^a^Chi-square test.

^b^two-sample *t*-test.
